# The gene regulatory role of non-coding RNAs in non-obstructive azoospermia

**DOI:** 10.3389/fendo.2022.959487

**Published:** 2022-08-18

**Authors:** Guanqing Zhou, Mimi Zhang, Jingzhi Zhang, Yaofeng Feng, Zhishen Xie, Siyi Liu, Detu Zhu, Yumei Luo

**Affiliations:** ^1^ Department of Obstetrics and Gynecology, Key Laboratory for Major Obstetric Diseases of Guangdong Province, The Third Affiliated Hospital of Guangzhou Medical University, Guangzhou, China; ^2^ Key Laboratory of Reproduction and Genetics of Guangdong Higher Education Institutes, The Third Affiliated Hospital of Guangzhou Medical University, Guangzhou, China; ^3^ Guangzhou Key Laboratory for Clinical Rapid Diagnosis and Early Warning of Infectious Diseases, Kingmed School of Laboratory Medicine, Guangzhou Medical University, Guangzhou, China

**Keywords:** non-obstructive azoospermia, non-coding RNA, microRNA, PIWI-interacting RNA, long non-coding RNA, circular RNA

## Abstract

Non-coding RNAs are classified as small non-coding RNAs, long non-coding RNAs and circular RNAs, which are involved in a variety of biological processes, including cell differentiation, proliferation, apoptosis and pathological conditions of various diseases. Many studies have shown that non-coding RNAs are related to spermatogenesis, maturation, apoptosis, function, etc. In addition, the expression of non-coding RNAs in testicular tissue and semen of patients with non-obstructive azoospermia was different. However, the role of non-coding RNAs in the pathogenesis of non-obstructive azoospermia has not been fully elucidated, and the role of non-coding RNAs in non-obstructive azoospermia is rarely reviewed. Here we summarize the research progress of non-coding RNAs in the pathogenesis of non-obstructive azoospermia.

## Introduction

8% of couples worldwide suffer from infertility, and 15% of infertile men are azoospermic, which can be divided into obstructive azoospermia (OA) and non-obstructive azoospermia (NOA) (1, 2). OA is caused by acquired factors and congenital abnormalities, such as infection, surgical trauma, congenital unilateral absence of vas deferens (CUAVD) and congenital bilateral absence of vas deferens (CBAVD) (3). NOA is a complex disease with high genetic heterogeneity and phenotypic heterogeneity caused by many factors, including chromosome abnormalities, Y chromosome microdeletions, gene mutations and epigenetic modifications (4). Non-coding RNAs (ncRNAs) play critical roles in gene regulation of etiology, which leads to azoospermia. NcRNAs are divided into two categories according to the size. The ones longer than 200 nucleotides (nt) are called long non-coding RNAs (lncRNAs), and those shorter than 200 nt are called small or short non-coding RNAs (sncRNA). (5). Among the sncRNAs, microRNA (miRNA) and PIWI-interacting RNA (piRNA) are widely studied in the reproductive system, which are important regulatory factors of gene expression in many cell pathways (6–8). LncRNAs also play important roles in cell differentiation, organogenesis, cellular homeostasis etc. (9–11). In addition, lncRNAs are involved in pathological states such as cancer, endocrine diseases and cardiovascular diseases (12–14). Circular RNA (circRNA), a single-stranded, covalently closed RNA molecule, is a newly discovered class of ncRNAs that plays a pivotal role in biogenesis, regulation, localization, degradation and modification (15, 16). All these ncRNAs are related to spermatogenesis, sperm maturation and sperm function. Here we summarize the research progress of ncRNAs in the pathogenesis of NOA.

## SncRNA

### microRNA

**Figure 1:**
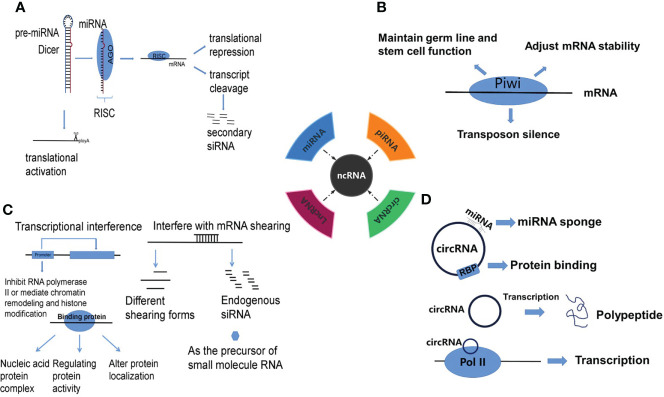
The mechanisms of gene regulation by **(A)** miRNA, **(B)** piRNA, **(C)** lncRNA and **(D)** circRNA.

**Figure 2:**
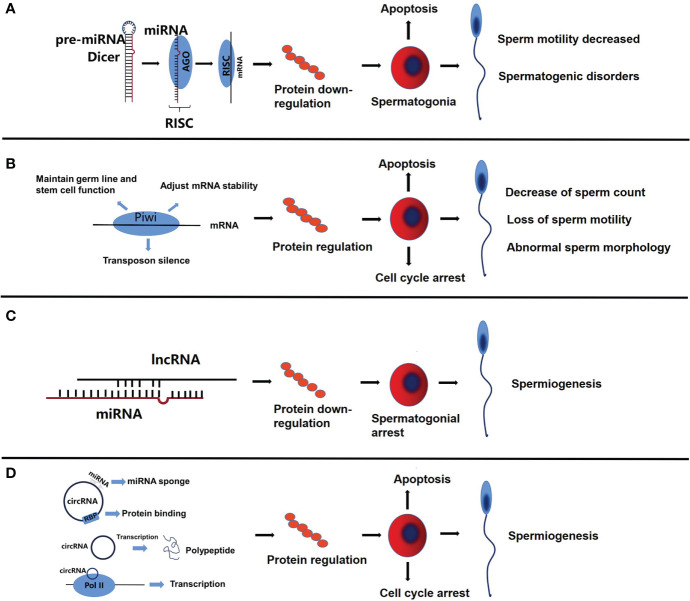
The functional roles of miRNA, piRNA, lncRNA and circRNA in sperms. **(A)** The complementary binding of miRNA and mRNA inhibits protein synthesis, which in turn promotes apoptosis of spermatogonia, decrease of sperm motility and spermatogenic disorders. **(B)** PiRNA can regulate the expression of related proteins, resulting in cell cycle arrest, decrease of sperm count, and the loss of sperm motility is related to abnormal sperm morphology. **(C)** LncRNA partially competes for miRNA binding sites, resulting in down-regulation of related proteins, which leads to spermatogonial arrest. **(D)** As a miRNA sponge, circRNA inhibits the function of miRNA, regulates protein binding and gene transcription, leads to cell cycle arrest and apoptosis of spermatogonia, and then affects spermatogenesis.

At present, substantial studies have shown that miRNA can lead to azoospermia through a variety of ways. There are 4 well-known routes of post-transcriptional gene regulation mediated by miRNAs: 1) translational repression, 2) translational activation, 3) transcript cleavage and 4) production of secondary siRNA (**Figure 1A**). Among these, translation inhibition is the most common mechanism in patients with NOA (17). Due to the incomplete complementarity required for binding, it is possible for a miRNA to target hundreds of different kinds of mRNAs (**Figure 2A**). By targeting the 3’-UTR of mRNAs, the expression of the targets is repressed, and then the subsequent biological functions of the targets are affected (18).

Spermatogenesis is a complex process that can be divided into 3 stages: 1) mitosis, 2) meiosis and 3) spermiogenesis (19). Defects in this process may lead to the occurrence of NOA, which is one of the causes of male infertility. MiRNAs are critical regulators of spermatogenesis. One study has compared 100 NOA patients with normal fertile men. The NOA patients showed significant overexpression of miR-141 and miR-7-1-3p in seminal plasma (20). The study found that the percentage of miR-141 methylation in NOA patients was significantly lower than that in fertile controls. And miR-141 down-regulated Cbl and Tgfb2, while miR-7-1-3p down-regulated Rb1 and Pik3r3, which was not conducive to spermatogenesis. In other studies, the low expression of miR-34c in NOA patient might affect spermatogenesis *via* deregulation of NOTCH2 (21, 22). And in another study, miR-34b/c and miR-449 double knockout (miR-dKO) male mice were infertile due to severe spermatogenic disorders and oligoasthenospermia. MiR-dKO sperm was injected into wild-type oocytes, led to a block at the two-pronucleus to zygote transition, which indicated that both miR-34b/c and miR-449 were essential for fertilization and preimplantation development, and the expression was down-regulated in infertile men (23, 24). MiR-122a affects the expression and transport of mRNAs and ncRNAs in germ cells by binding to Translin, and is down-regulated in NOA (24, 25). MiR-210 is highly expressed in the testis of NOA patients, which affects spermatogenesis by inhibiting the expression of NR1D2, and may be related to cryptorchidism as well (26). CDC25A is a spermatogenesis-related gene and the target gene of miR-15b. However the level of miR-15b in semen and plasma samples of NOA patients is significantly increased which can reduce the post-transcriptional activity of CDC25A gene and regulate spermatogenesis by targeting the 3’-UTR region of CDC25A (27).

Meiosis is an important process of spermatogenesis. Therefore, abnormal expression of meiosis-related proteins can lead to disturbance or abnormality of spermatogenesis. The stromal antigen 3 (STAG3) gene, which encoding a meiosis-specific cohesin component, is a strong candidate for male infertility. A study suggests that miR-3162-5p may lead to azoospermia by binding to the 3’UTR region of STAG 3 (28). Another study showed that the expression of miR-449 was significantly up-regulated at the beginning of meiosis during testicular development and adult spermatogenesis (29).

The increase of sperm apoptosis rate is one of the possible causes of azoospermia, and the abnormal expression of apoptosis-related proteins or genes may be related to the occurrence of azoospermia. The expression of miR-188-3p in testes of patients with azoospermia (NOA and OA) is down-regulated. The mechanism may be that down-regulation of miR-188-3p up-regulates the expression of MLH1 and promotes spermatogenic cell apoptosis by reducing histone acetylatio (30). A study of miRNA in the plasma of patients with NOA showed that the expression of miR-20a-5p was significantly up-regulated (31). Another study showed that miR-20a-5p was highly expressed in semen and promoted sperm apoptosis through STAT3 pathway (32). In addition, miR-19b is highly expressed in the semen of NOA, which affects spermatogenesis by affecting apoptosis (33). MiR-17-92 cluster (including miR-17, miR-18a, miR-19a, miR-20a, miR-19b-1 and miR-92a) is down-regulated in testicular tissue of NOA, which may down-regulate apoptotic genes, resulting in increased apoptosis in testes of NOA patients (34, 35).

Spermatogonial stem cells are a kind of cells that develop into spermatozoa and play an important role in maintaining high spermatogenesis (36). The expression of miR-135a in the semen of infertile patients decreased, and the molecules have semen specificity, the mechanism of infertility may reduce the number of spermatogonial stem cells by reducing the expression of FoxO1 (37, 38). This suggests that miR-135a seems to be beneficial to spermatogenesis. However, another study showed that the level of miR-135a was significantly negatively correlated with progressive sperm motility, and the mechanism may be that miR-135a-5p overexpression inhibits the expression of JAK2 protein by targeting the 3’-UTR of JAK2 mRNA (39).

Sertoli cells plays a key role in regulating spermatogenesis, while spermatogenesis is very low or absent in patients with Sertoli cell-only syndrome (SCOS) (40). In SCOS, the abnormal expression of miRNA may be the cause of NOA. MiR-133b and miR-202-3p are highly expressed in Sertoli cells of patients with SCOS. MiR-133b promotes the proliferation of human Sertoli cells by targeting GLI3, thus affecting spermatogenesis, while miR-202-3p controls the proliferation, apoptosis and synthesis of human Sertoli cells by targeting LRP6 and CCND1 of the Wnt/β-catenin signaling pathway (41, 42). However, another study showed that the down-regulated expression of miR-202-5p in Sertoli cells of SCOS patients was also associated with male gonadal development (43). Hsa-miR-320 is upregulated in testicular biopsies of patients with SCOS, which negatively regulates lactic acid production in Sertoli cells by directly inhibiting the expression of GLUT3. Lactic acid deficiency leads to Sertoli cell dysfunction, which leads to the loss of germ cells (44).

Other studies on spermatogenesis have shown that miR-192a is highly expressed in seminal plasma, and the mechanism of affecting spermatogenesis may be related to the activation of Caspase-3 protein and the regulation of genes related to testicular venous hypertension, hypoxia, increased oxidative stress and elevated body temperature in spermatic vein (45). In one study, it was found that the expression of miR-181c in NOA patients with successful sperm extraction was higher than that in patients with unsuccessful sperm extraction (46). Pri-mir-181c, the precursor of miR-181 c, is abundantly expressed in spermatozoa, but no mature form is observed in testis. Its maturation is mainly expressed after fertilization and can down-regulate the expression of CARM1. CARM1 is a key regulator of key pluripotent factors in human and mouse embryonic stem cells and blastomeres (47, 48). In a case-control study, miR-27a-3p was overexpressed in the testis of men with NOA, resulting in male infertility by directly down-regulating KDM3A and indirectly down-regulating TNP1 and PRM1 (49).

### PIWI-interacting RNA

Piwi interaction RNA(piRNA) is mainly expressed in germ cells, and its main mechanism is to maintain germ line and stem cell function, regulate the stability of translation and mRNA, and transposon silencing (**Figure 1B**), the aberration of which is related to cell cycle arrest, spermatogenesis, loss of sperm motility and abnormal sperm morphology in NOA (**Figure 2B**).

PiRNAs can modify the poly (A)-specific RNase-like domain containing 1 (PNLDC1)-like domain. When the area is destroyed, it will lead to azoospermia and male infertility (50, 51). Genes associated with the formation of piRNA may also lead to azoospermia. HIWI gene is very important for the biogenesis and function of piRNA. Its gene polymorphism leads to spermatogenic disorder, which may be a risk factor for male infertility (52). In addition, TDRD1 genetic polymorphism is involved in piRNA processing gene and is related to the risk of NOA in Han Chinese (53).

PiR_003399 exists in plasma and serum of mice. After treatment with microcystins leucine arginine, its expression level increases and has specific cytotoxic effect on spermatogonia. Inhibiting the expression of pir_003399 could increase the expression of CDK6 and significantly reduce the stagnation of cell cycle, decrease of mature sperm count, loss of sperm motility and abnormal sperm morphology induced by MC-LR (54). PiR_32362259 may affect the occurrence and development of spermatogenic injury in mice by regulating PI3K-AKT signal pathway. Its low expression can alleviate the decrease of cell viability, affect cell cycle and reduce the rate of apoptosis (55). PiR_121380 regulates the phosphorylation of ERK2 by targeting PTPN7, which affects sperm motility and fertility potential after thawing (56).

There are differences in the expression of piRNA in plasma, semen and testicular tissue between low fertility men and normal men, so this difference can be used as a biomarker for clinical diagnosis of male infertility. In another study, according to the success of NOA testicular sperm extraction, the experiment was divided into unsuccessful sperm extraction (USR) group and successful sperm extraction (SSR) group. The results showed that the expression of piRNAs was significantly down-regulated in 951 testes. 553 piRNA is completely absent in USR, but rich in SSR. Among them, 20 piRNA are involved in many important biological pathways, including apoptosis, cell proliferation and differentiation (57).In addition, seminal plasma piRNA was analyzed by high-throughput sequencing of semen from patients with NOA, asthenospermia and normal men. Five down-regulated piRNA (piR_31068, piR_31925, piR_43771, piR_43773, piR_30198) were screened from the semen of patients with NOA and asthenospermia, and piR_30198 can be used as a specific biomarker of azoospermia (58).

## LncRNA

LncRNA regulation includes epigenetic regulation of gene expression (DNA methylation, histone modification, chromatin remodeling), transcriptional regulation, and post-transcriptional regulation of gene expression (**Figure 1C**) (59, 60). Abnormal regulation of lncRNA in the genesis and development of spermatozoa might be one of the causes of NOA (**Figure 2C**).

Spermatogonia, the first stage of spermatogenesis and the early developmental stage of male germ cells (61). In recent years, studies have confirmed that lncRNA plays an important role in the proliferation, differentiation and apoptosis of spermatogonia (62–64). And there are similar reports in NOA. Meng Liang et al. found that lncRNAGm2044 and miR-202 were highly expressed in NOA of spermatogonial arrest, while Rbfox2 expression was inhibited (65). Their previous studies have shown that miR-202 can directly target RBFox2 and regulate spermatogenesis (66).

Competitive endogenous RNA (ceRNA) is one of the mechanisms of lncRNA action (67). LncRNA partially competes for miRNA binding sites, resulting in a decrease in miRNA level and damage in miRNA activity (**Figure 1C**). This mechanism also exists in patients with azoospermia (**Figure 2C**). Bo *et al.* used microarray to analyze the expression profile of LncRNA in NOA cells and identified 1036 differentially expressed lncRNAs. For examples, the expression of LINC00884, LINC00884, LEMD1-AS1 and ZFAS1 was up-regulated, while the expression of NSNK1G2-AS1, LINC00467, SPATA42 and ZNF295-AS1 was down-regulated. The lncRNA-miRNA-mRNA regulatory circuit suggests that lncRNA may participate in spermatogenesis *via* the ceRNA network. LINC00467 in this network regulates the expression of LRGUK and TDRD6 (68). In another study of lncRNA acting as ceRNA in patients with NOA, it was found that the expression of 74 mRNAs, 14 miRNAs and 10 lncRNAs was significantly different between the patient and the fertility group, and the expression of 10 lncRNAs was down-regulated. Among them, LINC00661, LINC00643, LINC00654, LINC00301, LINC00238, and LINC00905 were associated with miR-27-b-3p, and LINC00905, LINC00643, LINC00661, and LINC00654 were associated with miR-509-3-5p. MiR-509-5p and miR-27b-3p interact with target genes PLK1 and CRISP2, respectively (69).

Extracellular vesicles are a group of heterogeneous cell-derived membrane structures, which is now considered as an additional mechanism of cell-to-cell communication, allowing cells to exchange proteins, lipids and genetic materials (70). It plays an important regulatory role in tumors, cardiovascular diseases, infertility and other diseases. Now it is increasingly considered as a new way to find biomarkers (71–73). By comparing NOA with normal male semen plasma, Yun Xie et al. identified 9 specific Extracellular vesicles lncRNAs, including LOC100505685, SPATA42, CCDC37-DT,GABRG3-AS1, LOC440934, LOC101929088 (XR_927561.2), LOC101929088 (XR_001745218.1) and LINC00343 and LINC00301, suggesting that extracellular vesicle LncRNAs is a sensitive and specific method for predicting the presence of sperm in testis (74).

Testicular germinoma is the most common type of testicular cancer, in which cryptorchidism and male infertility are closely related entities. 88.6% of unoperated patients with cryptorchidism developed azoospermia (75). A study on azoospermia in cryptorchidism shows that gonadotropin-releasing hormone agonist treatment of cryptorchidism affects the expression of lncRNA in the testis. In this study, it is confirmed that LINC-ROR, LINC00221, LINC00261, LINC00282, LINC00293, LINC00303, LINC00898, LINC00994, LINC01121 and LINC01553 may play a role in the early stage of spermatogonial stem cell development (76).

In addition, genetic disease is also one of the diseases leading to azoospermia, autosomal or sex chromosome abnormalities can affect testicular spermatogenesis. The most common genetic disease that causes NOA is Klinefelter syndrome (KS), which is characterized by the presence of an extra X chromosome (77, 78). Winge *et al.* found that gonadotropins failed to differentiate into prospermatogonia in 8 fetal KS testes and 15 age-matched controls. Furthermore, transcriptome analysis of RNA sequencing from 4 fetal KS and 5 age-matched controls showed that there were 211 differentially expressed transcripts in fetal KS testis and enrichment of lncRNA (such as LINC01569 and RP11-485F13.1) in KS testis, suggesting that the failure of gonadotropin differentiation into spermatogonia may be due to the abnormal expression of lncRNA (79). Although cryptorchidism and Klinefelter syndrome have been confirmed to be associated with NOA, there are few studies on gene regulation and pathophysiology related to it, and further studies are needed.

## CircRNA

Circular RNA (circRNA) is a newly discovered class of ncRNA that can act as miRNA sponge, inhibit miRNA function, regulate protein binding and gene transcription, and have coding function (**Figure 1D**) (80). In NOA, circRNA, as a miRNA sponge, inhibits the function of miRNA, regulates protein binding and gene transcription, leads to cell cycle arrest and spermatogonia apoptosis, and then affects spermatogenesis (**Figure 2D**). More and more studies have shown that circRNA is related to NOA, mainly through competitive endogenous RNA network (ceRNA) to participate in the occurrence of NOA. One study identified 399 circRNA up-regulated and 1195 down-regulated by High throughput circRNA microarray analysis, constructed an up-regulated ceRNA of hsa-circRNA-101373 and identified multiple miRNA targets, which are involved in the processes of apoptosis, cell cycle arrest and spermatogenesis (81). Similarly, a study identified 19874 up-regulated circRNA and 18007 down-regulated circRNAs in the testis of patients with NOA by circRNA array, constructed a ceRNA network, and found that miRNAs paired with circRNAs is essential for the regulation of spermatogenesis (82). The expression of hsa-circRNA-0000116 in testicular tissue of patients with NOA is significantly higher than that in patients with OA, which affects fertility function through hsa_circ_0000116-miR-449-autophagy-related ceRNA network (83). Circ_0049356 participates in the regulation of actin cytoskeleton through circ_0049356-miRNA-mRNA pathway and plays an important role in the cytoskeleton rearrangement of germ cells during spermatogenesis (84). In addition, circRNA may regulate spermatogenesis by affecting the activity of SMAD protein in patients with NOA (85).

CircRNA has a significant differential expression in patients with NOA, so it can be used as a non-invasive molecular biomarker, therapeutic and drug target (86, 87).

## Discussion

In recent years, a large number of studies have deepened our understanding of the pathogenesis in NOA, most of which are regarding the molecular mechanisms mediated by ncRNAs, including miRNA, piRNA, lncRNA and circRNA. MiRNA plays a key role in the pathogenesis of NOA and is related to spermatogenesis, maturation and motility (88). This further proves that the abnormal expression or deletion of miRNA may play a key role in the occurrence of NOA. In this review, we found that most of the differentially expressed miRNA were up-regulated in NOA patients, and most of them were down-regulated targeting mRNA, which played a positive or negative role in spermatogenesis (**Table 1**). These miRNAs may be found in sperm, semen, testicular tissue and so on. Because there may be many or even hundreds of miRNA targets, it is impossible to treat the occurrence of sperm abnormalities by affecting downstream target genes. Therefore, miRNA should be the upstream target for the treatment of NOA (89). There have been studies to treat related diseases by regulating miRNA, through miRNA inhibitors, miRNA sponges, and through the use of miRNA mimics, or through the use of adeno-associated viruses (AAVs) to drive the expression of a given miRNA to restore miRNA levels (90). However, there is no publication on the treatment of NOA with miRNA targeting strategy. Similarly, there is no report of utilizing lncRNAs and circRNAs as therapeutic targets for NOA, despite many studies have proved that certain lncRNAs and circRNAs could be served as therapeutic targets for other diseases (91–95).

**Table 1:** Roles of miRNAs in azoospermia.

miRNA	Sample	Down/up-regulation	Mechanism	Effect on sperm	References
miR-141	Semen	Up-regulation	Downregulation of Cbl and Tgfb2	Negative	(20)
miR-7-1-3p	Semen	Up-regulation	Downregulation of Rb1 and Pik3r3	Negative	(20)
miR-449	Testis	Up-regulation	Downregulation of E2F	Positive	(29)
miR-34 c	Testis	Up-regulation	Downregulation of NOTCH2	Positive	(21)
miR-210	Testis	Up-regulation	Downregulation of NR1D2	Negative	(26)
miR-181 c	Zygote	Up-regulation	Downregulation of CARM1	Positive	(46–48)
miR-192a	Semen	Up-regulation	Activation of Caspase-3 protein	Negative	(45)
miR-20a-5p	Plasma and semen	Up-regulation	STAT3 Pathway	Negative	(31, 32)
miR-19b	Semen	Up-regulation	Indetermined	Negative	(33)
miR-27a-3p	Testis	Up-regulation	Downregulation of KDM3A	Negative	(49)
miR-133b	Sertoli cell	Up-regulation	Downregulation of GLI3	Positive	(41)
miR-202-3p	Sertoli cell	Up-regulation	Downregulation of LRP6 and CCND1	Negative	(42)
miR-320-3p	Sertoli cell	Up-regulation	Downregulation of GLUT3	Negative	(44)
miR-15b	Plasma and semen	Up-regulation	Downregulation of CDC25A	Negative	(27)
miR-122a	Spermatozoa	Down-regulation	Combine Translin	Negative	(24, 27)
miR-135a	Semen	Down-regulation	Downregulation of NR1D2 Downregulation of JAK2	Positive Negative	(38) (39)
miR-188-3p	Testis	Down-regulation	Upregulation of MLH1	Positive	(30)
miR-17-92	Testis	Down-regulation	Downregulation of apoptosis genes	Positive	(35)

PiRNA is mainly expressed in germ cells, and many studies have confirmed that piRNA is related to spermatogenesis, loss of sperm motility and abnormal sperm morphology (96–98). Therefore, piRNA is an excellent study object in the area of NOA. This review has shown that piRNA is related to spermatogenesis and motility, and can affect sperm motility after thawing, so it is a potential therapeutic target. In the view of the particularity of piRNA expression site, it seems to have the potential of specific therapeutic targets. In addition, some researchers have screened out specific biomarkers of azoospermia by analyzing piRNA in seminal plasma, which is a biomarker for clinical diagnosis of male infertility.

Current studies have shown that there is a difference in the expression of lncRNA between NOA patients and normal men. On one hand, it can function as the host gene of miRNAs, indirectly regulating the expression of the target proteins, thus regulating spermatogenesis. In addition, lncRNA can also function as a ceRNA, through partial complementary competitive binding sites with miRNA, resulting in a decrease in the level of miRNA and impairment of miRNA activity, and then regulate spermatogenesis and maturation. However, other gene regulatory mechanisms of lncRNA, such as chromatin remodeling and transcriptional regulation, are lacking in NOA research (99). To this end, it is necessary to explore other mechanisms of action of lncRNA in NOA. In addition, studies on cryptorchidism and Klinefelter syndrome have shown that lncRNA can affect the development and differentiation of spermatogonial stem cells. Extracellular vesicle lncRNA is sensitive and specific and can be used as a marker for predicting the presence of sperm in the testis, suggesting that lncRNA can be used as a diagnostic marker for patients with NOA.

Since Hansen *et al.* reported the functional analysis of naturally expressed circRNAs (100), circRNAs have attracted great concerns from researchers. CircRNAs are differentially expressed in NOA patients, which mainly regulates spermatogenesis and apoptosis through the circRNA-miRNA-mRNA regulatory circuit.

The role of ncRNA in the pathogenesis of NOA has not been clearly clarified; hence, there is a need of more comprehensive investigation on lncRNA, circRNA, miRNA and piRNA in the field of spermatogenesis.

## Author contributions

DZ and YL conceived the study. GZ and MZ prepared the figure and table. GZ, MZ, JZ, ZX, SL, YF, DZ and YL wrote and edited the manuscript. All authors read and approved the final manuscript.

## Funding

This research was funded by the National Natural Science Foundation of China (82002774), Guangdong Provincial Natural Science Foundation (2020A1515010065), Guangzhou City Science, Technology and Innovation Commission (201804010340, 202002030077), Guangzhou City Science and Technology Planning Project (202201020208), Guangdong Province Outstanding Youth Medical Talent Program (110217110) and Lin He’s Academician Workstation of New Medicine and Clinical Translation at The Third Affiliated Hospital of Guangzhou Medical University (2021HLKY05).

## Conflict of interest

The authors declare that the research was conducted in the absence of any commercial or financial relationships that could be construed as a potential conflict of interest.

## Publisher’s note

All claims expressed in this article are solely those of the authors and do not necessarily represent those of their affiliated organizations, or those of the publisher, the editors and the reviewers. Any product that may be evaluated in this article, or claim that may be made by its manufacturer, is not guaranteed or endorsed by the publisher.
